# Time to Local Recurrence as a Predictor of Survival in Patients With Soft Tissue Sarcoma of the Extremity and Abdominothoracic Wall

**DOI:** 10.3389/fonc.2020.599097

**Published:** 2020-11-04

**Authors:** Yao Liang, Tianhui Guo, Dongchun Hong, Wei Xiao, Zhiwei Zhou, Xing Zhang

**Affiliations:** ^1^ State Key Laboratory of Oncology in South China, Collaborative Innovation Center for Cancer Medicine, Guangzhou, China; ^2^ Department of Gastric Surgery, Sun Yat-sen University Cancer Center, Guangzhou, China; ^3^ Department of Medical Melanoma and Sarcoma, Sun Yat-sen University Cancer Center, Guangzhou, China

**Keywords:** time to local recurrence, soft tissue sarcoma, extremity and abdominothoracic wall, survival, prognostic factors

## Abstract

**Objective:**

The purpose of this retrospective study was to identify the prognostic significance of time to local recurrence (TLR) with regard to overall survival (OS) and survival after local recurrence (SAR) in patients with soft tissue sarcoma (STS) of the extremity and abdominothoracic wall.

**Methods:**

We identified 477 patients who underwent R0 resection for localized STS of the extremity and abdominothoracic wall, from January 1995 to December 2016, of whom 190 patients developed local recurrence as their first recurrent event. Based on TLR, patients were divided into two groups: early local recurrence (ELR, <12 months) and late local recurrence (LLR, ≥12 months). The Kaplan–Meier method and Cox regression analysis were used to estimate the OS and SAR, and to identify factors associated with patient outcomes.

**Results:**

The median follow-up time for the entire cohort was 118.4 months, and was 118.5 months for the 190 patients who developed local recurrence. Deep tumor location (HR 1.73, 95% CI 1.27–2.37, P = 0.001) and tumor grade ≥2 (G2 vs. G1: HR 1.75, 95% CI 1.21–2.53, G3 vs. G1: HR 2.57, 95% CI 1.66–3.98, P < 0.001) were associated with a higher rate of local recurrence. There were 99 patients in the ELR group and 91 in the LLR group, with a median TLR of 10.8 months for the entire cohort. Patients from the ELR group had a shorter OS and a lower 5-year OS rate than the LLR group. Univariate and multivariate analyses demonstrated TLR as an independent prognostic factor for SAR and OS, in addition to tumor grade. Also, surgical treatment and absence of metastasis after local recurrence were associated with longer SAR.

**Conclusions:**

In patients with STS of the extremity and abdominothoracic wall, ELR after R0 resection indicated a worse prognosis than those with LLR, and TLR can be considered an independent prognostic factor for OS and SAR. Furthermore, local recurrence was significantly influenced by the depth and the histopathological grading of the primary tumor, and reoperation after local recurrence could improve survival, which means salvage surgery may still be the preferred treatment when there are surgical indications after recurrence.

## Introduction

Soft tissue sarcomas (STSs) are a heterogeneous group of malignancies with a low incidence, accounting for approximately 1% of all adult malignancies (1). STSs may arise in different body sites, including the head or neck, extremity, trunk, retroperitoneum, or chest wall, with local aggressiveness. Among all of STS, about 80% of tumors locate in the extremities and superficial trunk. There are more than 50 different histologic subtypes identified, each with distinct biologic behavior and clinical manifestation. The anatomic sites and pathologic subtypes of these tumors are crucial for their treatments and outcomes. Despite the established role of radical or wide surgical resection as a standard of treatment, 15%–40% of patients with localized STS tumors develop recurrence and have a dismal 5-year survival rate ranging between 55% and 70% (2, 3). Thus, tumor local relapse remains one of the major problems in managing STS, and can be defined as early or late recurrence. In breast adenocarcinoma, renal cell carcinoma, and gastric cancer, it was previously reported that patients with late recurrence had better prognosis than those with early recurrence (4–6). However, to the best of our knowledge, neither significant factors affecting the survival after recurrence (SAR) for STS patients nor information concerning the prognostic significance of time to local recurrence (TLR) in STS patients have been reported.

Therefore, we performed this retrospective study to determine the clinicopathological factors affecting local recurrence (LR), and the prognostic significance of TLR, with regard to overall survival (OS) and SAR, in patients with STS of the extremity and abdominothoracic wall.

## Methods

### Study Population

The data of 769 patients who underwent R0 resection for primary STS at the Sun Yat-sen University Cancer Center (SYSUCC, Guangzhou, China), from January 1995 to December 2016, were retrieved. As there is no clear standard for defining radical or extensive resection of STS, due to the existing different tumor types, tumor volume, and location, here, we used the standardized classifications (R0, R1, R2) of the International Union Against Cancer (UICC) for surgery to classify the radicality of the surgical resections performed (7). R0 was defined as the microscopic absence of malignant cells at the resection margin. Patients with R1 or R2 resection were excluded as they comprised of a very small proportion of the retrieved cases. Seventy-seven of the 769 (10%) patients were lost to follow-up and were excluded. Patients with inadequate medical records (5 patients) and distant metastasis at the time of initial diagnosis (82 patients) were also excluded. Although the proportion is low, patients who received the adjuvant treatments (23.9%), including chemotherapy (mostly doxorubicin-based), radiotherapy, or chemoradiotherapy, were included for the analysis. All adjuvant treatments were planned based on the patients’ disease stage and willingness to abide to treatment, and the regimen prescribed was based on the treating oncologist’s discretion. Finally, 477 patients were included in this study (**Additional File 1: Figure S1**).

Local recurrence was defined as tumor relapse in the operative field following R0 resection according to follow-up radiographic evidence, physical exam, or self-reported symptoms. Among the 477 patients, 190 patients were diagnosed with local recurrence as their first recurrent event, which was then histologically confirmed. Most of the patients with local recurrences underwent secondary resection, except for a small percentage of patients who received chemotherapy (n = 6) or radiotherapy (n = 1) only. The 190 patients were then classified into two groups according to their TLR, which was calculated from the date of R0 resection to the date of initial local recurrence. Patients who were diagnosed with TLR within 12 months (n = 99) were grouped into an early local recurrence (ELR) group while those diagnosed with TLR no less than 12 months (n = 91) were included in a late local recurrence (LLR) group. As there is no standard definition for early and late local recurrence, the 12 months cutoff value was determined based on published literatures (8, 9).

This study was approved by the institutional review board of SYSUCC (No. B2020-008-01), and the ethics committee waived the need for informed consent as this was retrospective study. All patients’ data used was anonymously analyzed.

### Data Collection

Clinical and pathological data of the included patients were retrospectively obtained from the patient’s medical records. Tumor stage was classified using the AJCC 8th Edition (10), and the tumors were graded according to the Fédération Française des Centres de Lutte Contre le Cancer (FNCLCC) grading system (11).

The authenticity of this article was validated by uploading the key raw data to the Research Data Deposit public platform (www.researchdata.org.cn) with the approval RDD number of RDDA2019001332.

### Follow-Up

All patients were routinely followed with physical examination, computerized tomography or magnetic resonance imaging every 3 to 6 months for the first 2 years after resection, then annually *via* outpatient visits or telephone interviews by the independent follow-up department of SYSUCC. The minimum follow-up time was 6 months. The final survival follow-up time was considered the latest follow-up date of this study (October 1, 2019) or death. OS was defined as the time between the R0 resection and death of any cause or the last follow-up. SAR was defined as the time from the date of diagnosis of local recurrence to the last follow-up date or the date of death.

### Statistical Analysis

The chi-square test of independence was used to test the distributive correlations between the clinicopathological variables and local recurrence. Survival curves were analysed by Kaplan-Meier method, and differences between survival rates were compared by using the log-rank test (12). The Cox proportional hazard model with the stepwise forward selection algorithm was used to find out independent prognostic variables associated with LR, OS, and SAR, and the results are presented as hazard ratios (HR) and 95% confidence intervals (95% CI). Two-sided *P* values < 0.05 were considered statistically significant. All data were analyzed using the IBM SPSS software, version 20.0 (SPSS, Inc., and IBM Company, Armonk, New York).

## Results

### Baseline Patient Characteristics

The patients’ baseline characteristics are shown in **Table 1**. The median age of the 477 patients was 42 years (range: 6–85 years). There were 284 male patients and 193 female patients in a ratio of 1.47:1. Fibrosarcoma (137, 28.7%) and undifferentiated pleomorphic sarcoma (104, 21.8%) were the most common pathological types. G1 tumors were identified in 135 (28.3%) patients, G2 in 226 (47.4%) patients, and G3 in 72 (15.1%) patients. Most patients had stage II disease (177, 37.1%). In addition, 135 (28.3%) patients had stage I disease and 121 (25.4%) had stage III disease. Due to the lack of understanding of the disease and standard treatment, only a small percentage of the STS patients received postoperative therapy, including chemotherapy (28, 5.9%), radiotherapy (67,14.0%) and chemoradiotherapy (19, 4.0%), spanning a period of 21 years. By comparisons, patients with deep tumor depth, G2-G3 tumor grade and II-III AJCC stage are more likely to receive adjuvant therapy (all P < 0.001).

**Table 1:** >Baseline characteristics of the entire study cohort (n = 477).

Characteristics	Cases	Percentage (%)
Sex	477	
Male	284	59.5
Female	193	40.5
Age at operation (years)		
<50	300	62.9
≥50	177	37.1
Body mass index (kg/m^2^)		
<18.5	53	11.1
≥18.5 to <25.0	299	62.7
≥25.0	125	26.2
Pathological types		
Fibrosarcoma	137	28.7
Liposarcoma	65	13.6
Undifferentiated pleomorphic sarcoma/MFH	104	21.8
Leiomyosarcoma	12	2.5
Synovial sarcoma	63	13.2
Rhabdomyosarcoma	19	4.0
Alveolar soft part sarcoma	6	1.3
Angiosarcoma	6	1.3
Malignant peripheral nerve sheath tumor	31	6.5
Mesenchymal chondrosarcoma	14	2.9
Others	20	4.2
Tumor size (cm)		
<5	262	54.9
≥5	215	45.1
Tumor site		
Upper extremity	117	24.5
Lower extremity	182	38.2
Thoracic/trunk/abdominal wall	178	37.3
Tumor depth		
Superficial	211	44.2
Deep	266	55.8
Tumor grade		
G1	135	28.3
G2	226	47.4
G3	72	15.1
Missing	44	9.2
AJCC stage		
IA	91	19.1
IB	44	9.2
II	177	37.1
IIIA	91	19.1
IIIB	30	6.3
Missing	44	9.2
End-point		
Alive	404	84.7
Dead	73	15.3
Local Recurrence		
Yes	190	39.8
No	287	60.2
Metastasis after recurrence		
Yes	54	11.3
No	423	88.7
Adjuvant therapy		
None	363	76.1
Chemotherapy	28	5.9
Radiotherapy	67	14
Combined chemoradiotherapy	19	4
Therapy after recurrence		
None	7	3.7
Surgery alone	108	56.8
Chemotherapy alone	6	3.2
Radiotherapy alone	1	0.5
Surgery + Chemotherapy	23	12.1
Surgery + Radiotherapy	24	12.6
Surgery + chemoradiotherapy	17	8.9
Combined chemoradiotherapy	3	1.6
Radiofrequency	1	0.5

### Local Recurrence Rate and Influencing Factors

**Figure 1:**
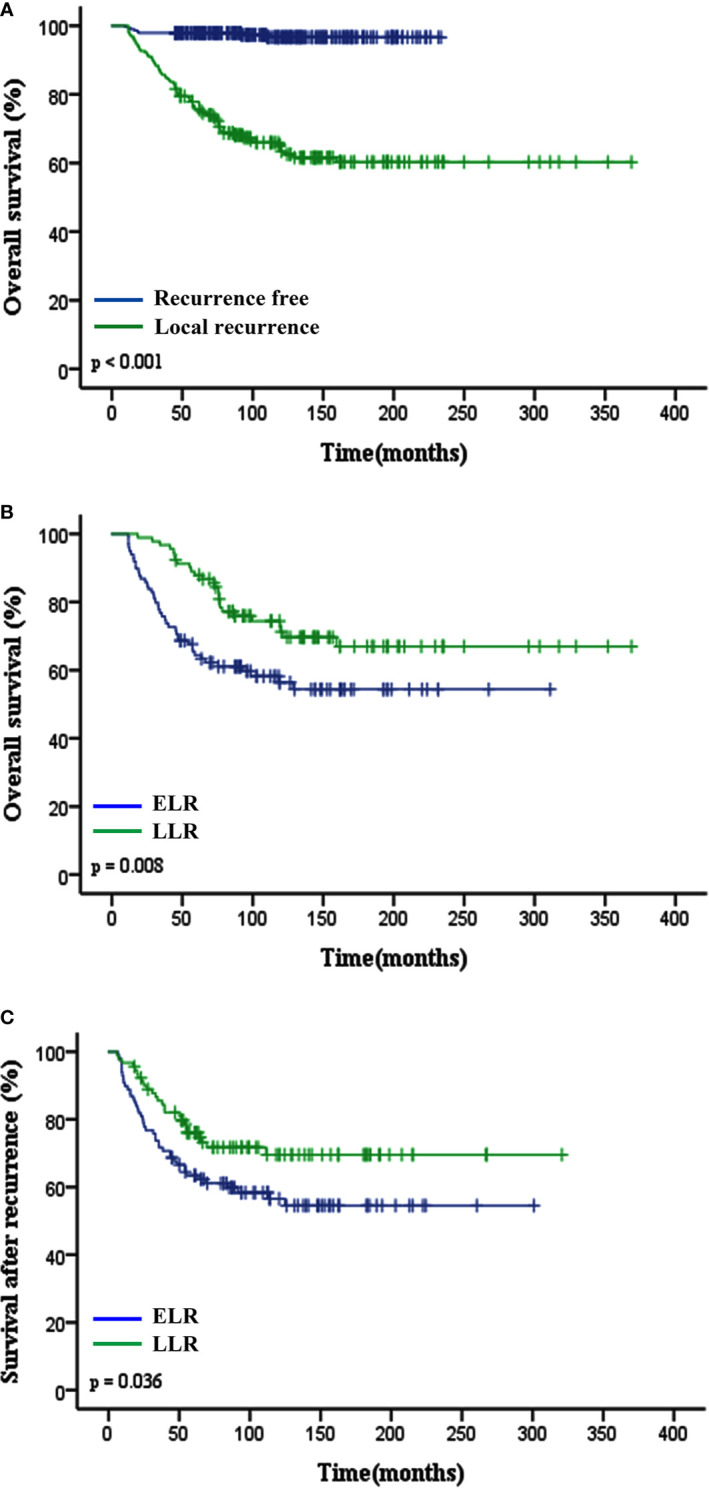
Impact of local recurrence and TLR (ELR vs. LLR) on clinical outcomes of patients with STS of extremity and abdominothoracic wall. **(A)** Overall survival in recurrent free and local recurrent patients (p < 0.001). **(B)** Overall survival (p = 0.008) and **(C)** Survival after recurrence (p = 0.036) curves showed that patients in the ELR group had a worse prognosis than those in the LLR group. TLR, time to local recurrence; ELR, early local recurrence (<12 months after primary surgery); LLR, late local recurrence (≥12 months after primary surgery).

Over a median follow-up time of 118.4 months (range 9.6–368.8 months), 73 (15.3%) patients died, and 190 (39.8%) experienced local recurrence. Fifty-four (28.4%) of the 190 patients with local recurrence developed distant metastasis. A total of 46 patients had grade 3 sarcomas, 105 had grade 2, and 39 had grade 1. In 61 patients the depth of the tumor was superficial and in 129 it was deep. The recurrence rates observed in patients classified as stage I, II, and III were 29.6% (40/135), 44.1% (78/177), and 59.5% (72/121), respectively. However, there were no differences in the incidence of local recurrence between the patients with or without postoperative treatments (P = 0.096), and this might be on account of the small sample. Histological subtype (e.g., fibrosarcoma, undifferentiated pleomorphic sarcoma, liposarcoma and synovial sarcoma) did not affect the local recurrence in this study. Furthermore, the 5- and 10-year OS rates of patients who did not develop local recurrence were significantly higher than those who developed local recurrence (97.9% vs. 75.7%; 96.6% vs. 63.4%; p < 0.001; **Figure 1A**)****. Deep tumor location (deep vs. superficial: HR 1.73, 95% CI 1.27–2.37, P = 0.001) and tumor grade ≥ 2 (G2 vs. G1: HR 1.75, 95% CI 1.21–2.53, G3 vs. G1: HR 2.57, 95% CI 1.66–3.98, P < 0.001) were significantly associated with a higher rate of local recurrence (**Table 2**).

**Table 2:** Univariate and multivariate analyses of variables for local recurrence in STS patients.

Variables	LR Univariate analysis		LR Multivariate analysis	
	HR (95% CI)	p value	HR (95% CI)	p value
Sex		0.416		
Male	1 (referent)			
Female	0.89 (0.66–1.19)			
Age (years)		0.048		
<50	1 (referent)			
≥50	1.34 (1.00–1.79)			
Tumor size (cm)		0.007		
<5	1 (referent)			
≥5	1.48 (1.12–1.97)			
Tumor depth		<0.001		0.001
Superficial	1 (referent)		1 (referent)	
Deep	1.95 (1.43–2.64)		1.73 (1.27–2.37)	
Tumor Grade		<0.001		<0.001
G1	1 (referent)		1 (referent)	
G2	1.88 (1.30–2.72)		1.75 (1.21–2.53)	
G3	3.08 (2.01–4.73)		2.57 (1.66–3.98)	
AJCC stage		<0.001		
IA + IB	1 (referent)			
II	1.69 (1.15–2.47)			
IIIA + IIIB	1.79 (1.83–3.97)			
Adjuvant therapy		0.111		
Yes	1 (referent)			
No	0.77 (0.56–1.06)			

LR, local recurrence; HR, hazard ratio; CI, confidence interval.

### Association Between TLR and Survival

The median TLR was 10.8 months (range 1.4–190.7 months). Patients in the ELR group had a shorter median OS time and lower 5-year OS rate than those in the LLR group (P = 0.008; 64.4% vs. 87.9%, P < 0.001; **Figure 1B**). Patients with LLR had a longer SAR than patients with ELR (P = 0.036; **Figure 1C**).

**Figure 2:**
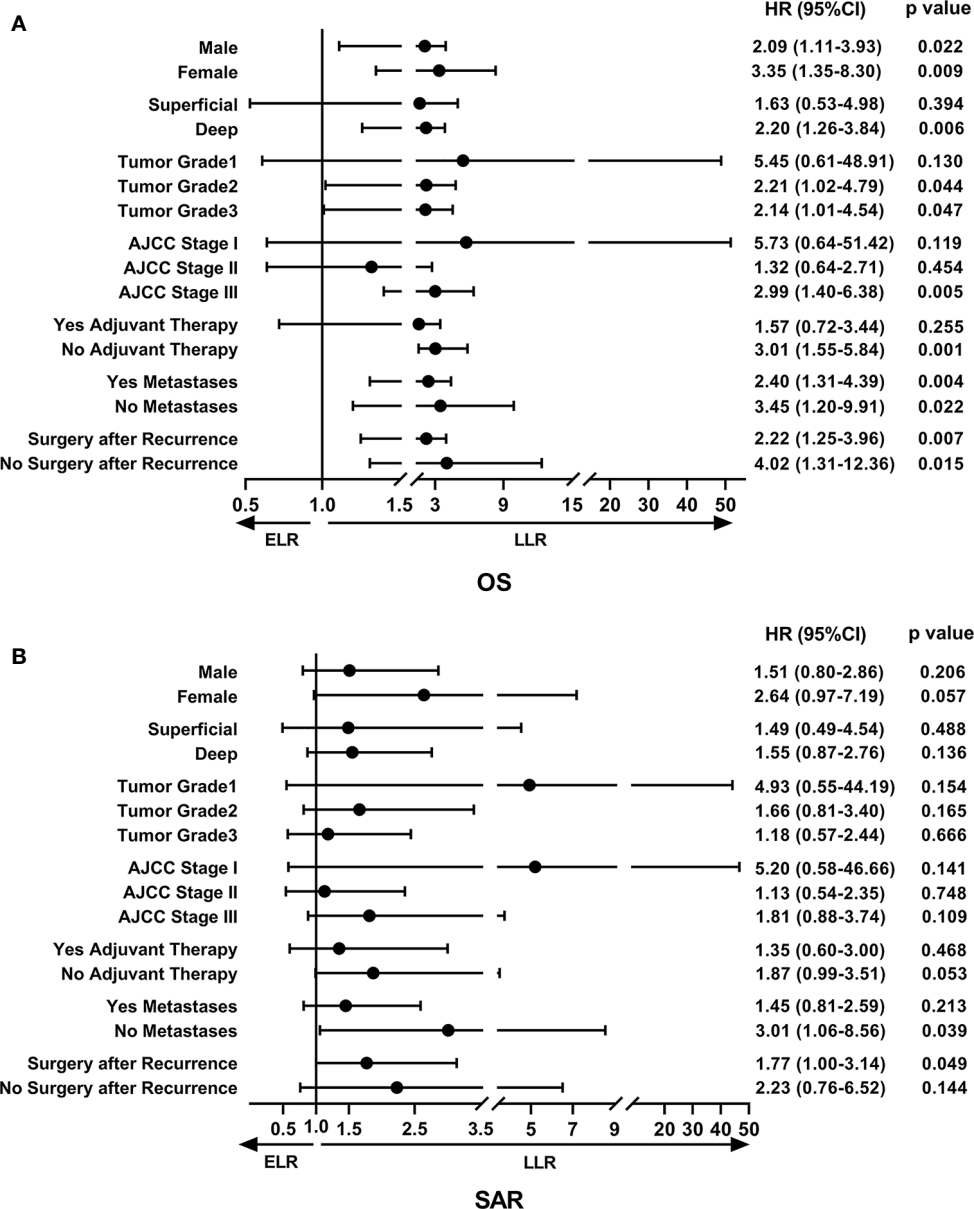
The forest plot of prognostic relevance of TLR and relevant clinicopathological parameters using the Cox proportional hazards model. **(A)** Overall survival curve showed that there were significant differences between the ELR and LLR groups for patients with tumor grade ≥ 2, stage III disease and without adjuvant therapy after initial R0 surgery. **(B)** Survival after recurrence curve showed that patients without metastases or with surgery after local recurrence in the LLR group exhibited a better SAR than those in the ELR group. OS, overall survival; SAR, survival after recurrence; ELR, early local recurrence (<12 months after primary surgery); LLR, late local recurrence (≥12 months after primary surgery); HR, hazard ratio; CI, confidence interval.

To determine the factors affecting the prognosis of ELR and LLR patients, the prognostic relevance of TLR and the patients’ clinicopathological parameters were analyzed using the Cox proportional hazards model (**Figure 2**). Our results demonstrated that LLR patients had better OS than ELR patients in both gender (male, female), the presence of metastasis, and the performance of surgery after local recurrence. Furthermore, there were significant differences in OS between the two groups for patients with tumor grade ≥ 2 (G2: HR 2.21, 95% CI 1.02–4.79, P = 0.044; G3: HR 2.14, 95% CI 1.01–4.54, P = 0.047), with stage III disease (HR 2.99, 95% CI 1.40–6.38, P = 0.005), and without adjuvant therapy after initial R0 surgery (HR 3.01, 95% CI 1.55-5.84, P = 0.001) (**Figure 2A**).

In addition, there were no statistically significant differences in SAR between the two groups regardless of sex, tumor depth, tumor grade, AJCC stage, and adjuvant therapies. However, it was worth noting that patients without metastases (HR 3.01, 95% CI 1.06–8.56, P = 0.039) or with surgery (HR 1.77, 95% CI 1.00–3.14, P = 0.015) after local recurrence in the LLR group exhibited a better SAR than those in the ELR group (**Figure 2B**).

Multivariate analyses revealed that TLR and tumor grade were independent prognostic factors for both OS (P = 0.014, P < 0.001) and SAR (P = 0.006, P = 0.022). Moreover, for the 190 patients with local recurrence, non-surgical treatment and metastases after recurrence were negative prognostic factors for SAR, with HRs of 1.94 (95% CI 1.06–3.57, P = 0.033) and 0.12 (95% CI 0.07–0.23, P < 0.001), respectively (**Tables 3** and **4**).

**Table 3:** Univariate and multivariate analyses of variables for overall survival in STS patients.

Variables	OS Univariate analysis		OS Multivariate analysis	
	HR (95% CI)	p value	HR (95% CI)	p value
Sex		0.531		
Male	1 (referent)			
Female	1.16 (0.74–1.82)			
Age (years)		0.534		
<50	1 (referent)			
≥50	1.16 (0.73–1.83)			
Tumor size (cm)		0.002		
<5	1 (referent)			
≥5	2.05 (1.29–3.26)			
Tumor depth		<0.001		
Superficial	1 (referent)			
Deep	2.80 (1.65–4.76)			
Tumor Grade		<0.001		<0.001
G1	1 (referent)		1 (referent)	
G2	5.20 (2.02–13.38)		2.98 (1.17-7.67)	
G3	8.95 (3.51–22.82)		9.04 (3.50-23.34)	
AJCC stage		<0.001		
IA + IB	1 (referent)			
II	4.33 (1.70–11.06)			
IIIA + IIIB	16.79 (6.53–43.15)			
Recurrence		<0.001^a^ 0.009^b^		<0.001^a^ 0.014^b^
Free	1 (referent)		1 (referent)	
LLR	9.96 (4.47–21.79)		13.03 (4.53–37.48)	
ELR	18.67 (8.76–39.78)^a^ 1.92 (1.18–3.13)^b^		23.90 (8.53–67.00)^a^ 1.85 (1.13-3.02)^b^	
Adjuvant therapy		0.039		
Yes	1 (referent)			
No	0.61 (0.38–0.98)			

a Recurrence-free group as the referent;

b Late local recurrence group as the referent.

OS, overall survival; HR, hazard ratio; CI, confidence interval; LLR, late local recurrence; ELR, early local recurrence.

**Table 4:** Univariate and multivariate analyses of variables for SAR in STS patients.

Variables	SAR Univariate analysis		SAR Multivariate analysis	
	HR (95% CI)	p value	HR (95% CI)	p value
Sex		0.400		
Male	1 (referent)			
Female	1.23 (0.76–2.00)			
Tumor depth		0.006		
Superficial	1 (referent)			
Deep	2.34 (1.28–4.30)			
Tumor Grade		<0.001		0.006
G1	1 (referent)		1 (referent)	
G2	2.58 (1.01–6.63)		0.97 (0.35–2.72)	
G3	8.55 (3.31–22.08)		2.26 (0.77–6.58)	
AJCC stage		0.006		
IA + IB	1 (referent)			
II	3.54 (1.37–9.15)			
IIIA + IIIB	4.60 (1.80–11.80)			
TLR		0.038		0.022
LLR	1 (referent)		1 (referent)	
ELR	1.69 (1.03–2.77)		1.79 (1.09–2.95)	
Adjuvant therapy		0.032		
Yes	1 (referent)			
No	1.72 (1.05–2.82)			
Metastasis after recurrence		<0.001		<0.001
Yes	1 (referent)		1 (referent)	
No	0.09 (0.05–0.15)		0.12 (0.07–0.23)	
Therapy after recurrence		<0.001		0.033
Surgery	1 (referent)		1 (referent)	
No surgery	5.06 (2.87–8.93)		1.94 (1.06–3.57)	

SAR, survival after recurrence; HR, hazard ratio; CI, confidence interval; LLR, late local recurrence; ELR, early local recurrence.

## Discussion

Local recurrence is a common reason for treatment failure after R0 surgery in STS (13). To assess the effects of local recurrence and other clinicopathological factors on survival, especially TLR, in patients with STS of the extremity and abdominothoracic wall, we performed a retrospective study based on data from 477 patients from the SYSUCC. This study is—to our best knowledge—the largest study to analyze the association between the TLR and survival in patients with STS.

In this study, a high local recurrence rate was found in STS (n = 190, 39.8%), which was slightly above other relevant literatures (14–16). There can be several reasons for this. First, although the tumor treatment condition has been improved in the past years in China, patients usually come to the hospital in a comparatively later stage or when their symptoms have been aggravated. Most of the patients included in this study had advanced tumor grade (G2-G3) or stage (II-III) with deep location at the time of initial diagnosis. And also, the percentage (only 23.9%) of patients who received postoperative adjuvant treatment was low due to clinical, financial, or personal reasons. Second, all of the patients included in this study had received the R0 resection, but the distance between the tumor and the surgical margins was not clear completely owing to the long retrospective span. As we all know that those patients who presented with a surgical margin of 2 mm or less might have a worse survival and a higher local recurrence rate (9). Moreover, this article involved a cohort of patients with long follow-up time (some for more than 15 years), based on which the risk for local recurrence was observed to increase accordingly.

Tumor recurrence is a well-known factor for poor prognosis of STS. Zhao et al. (17) and Eilber et al. (18) reported respectively in 133 and 753 STS patients groups that there was a lower 5-year OS rate in the local recurrence group than in the no local recurrence group. Posch et al. (19) observed that patients with local recurrence were more likely to develop distant metastasis (HR = 8.4; 95% CI, 4.3–16.5; P < 0.001). Another study demonstrated that 17% of patients with extremity STS after R1 resection died of local recurrence without any distant metastasis (20). All of these studies illustrated that local recurrence had a negative effect on the survival of patients with STS, which was in accordance with our study findings.

Some investigators reported that prognostic factors such as surgical margin and location played an important role in local control and were associated with the local recurrence in STS (15, 21, 22). In addition, high tumor grade, larger tumor size, and deep tumor location were also considered as predictors of local recurrence in STS (17, 19, 23, 24). Consistent with other studies (20), tumor depth and tumor grade were identified as significant prognostic factors affecting local recurrence by multivariate analysis in our study. Since early diagnosis of STS recurrence is important to offer the patient a realistic second treatment chance, an adequate identification of patients at higher risk, those with a deep tumor location and higher tumor grade, can promote the development of individualized surveillance programs. These patients may require more extensive resection and closer postoperative follow-up, and may be considered for additional preoperative therapy or more intense adjuvant chemotherapy to reduce the risk of recurrence. However, AJCC stage were not independent predictors of local recurrence, OS and SAR in our analysis, which is similar to a previous large-scale study (25). This could be due to the staging defects of human subjectivity and the heterogeneity of STS. The 8th AJCC stage system illustrated an unprecedented change for risk stratification by redefining the T-stage categories (26), which disregarding the independent prognostic information provided by tumor depth. Superficial tumors are associated with better outcomes than deep ones, even after controlling for tumor size and histologic grade (27, 28). Our data suggest that the system still needs further investigation to improve risk stratification.

TLR as a predictior for survival in patients with various cancers has been researched with divergent results. Several studies have suggested that TLR is a prognostic factor for survival in primary breast sarcoma, renal cell carcinoma and gastric cancer (4, 6, 29), while others have not found TLR of significant importance (8, 30). Sugiura et al. found the survival rate was lower in STS patients with local recurrence developing within 2 years than after 2 years (46% vs. 83%, P = 0.01) (31). Our study confirmed that TLR in patients with STS of the extremity and abdominothoracic wall was associated with survival and was considered an independent prognostic factor for OS and SAR, and patients with ELR (TLR within 12 months) indicated worse prognosis compared with those with LLR (TLR no less than 12 months). In addition, our research included 54 patients who developed distant metastasis after the local recurrence. We found that patients without metastases after local recurrence in the LLR group also exhibited a better SAR than those in the ELR group, but there was no difference in patients with distant metastases between two groups. The study from Posch (19) demonstrated that patients who suffered a local recurrence were more likely to develop distant matastasis and patients with distant metastasis after a long tumor-free interval did not show a better survival prognosis compared to those with distant metastasis occurring early after primary surgery, which had similar views with our research.

Moreover, though there is no concrete proof, it is generally accepted that STS patients with local recurrence need another resection with a goal of negative margins. A previous study reported that local recurrence in retroperitoneal STS patients was amenable to surgery, which could improve survival (32). Our results confirmed that operation after recurrence was also strongly associated with better SAR in STS of the extremity and abdominothoracic wall, indicating that salvage surgery may still be the preferred treatment when there are surgical indications after recurrence. This observation supports the mainstream view nowadays.

There were some limitations of the analyses in this study that should be noted. First, this was a retrospective study which could have inherent sources of transfer bias (i.e., loss to follow-up) and selection bias (i.e., clinical decision based on economic condition by patients), and we enrolled consecutive patients to reduce the influence of possible selection bias. Second, all patients enrolled in this study were selected from one hospital, the SYSUCC and therefore, the investigated patients’ characteristics and the study results may not be generalizable to other populations. Our conclusions should be verified in a larger population of STS patients from multiple centers. In addition, the clinicopathological data of some patients, such as data on AJCC stage in 44 patients, and the details of adjuvant therapy, were incomplete owing to the huge spans of time, thus we were unable to provide more information about the effects of the chemotherapy and/or radiotherapy. It seems reasonable that tumors with different subtypes may exhibit different clinical behaviors and altered survivals, and this is a topic that requires further investigation to figure out the relationship between the histologic subtypes and local recurrence.

Despite these limitations, this is the largest study based on a heterogeneous group of patients to demonstrate the prognostic values of TLR in STS of the extremity and abdominothoracic wall, the conclusions postulated remain highly reasonable.

## Conclusion

Our results showed that local recurrence was significantly associated with a decreased OS in patients with STS of the extremity and abdominothoracic wall, and those with deeply located initial tumor or a higher tumor grade were more likely to experience local recurrence than their counterparts. Surgery after local recurrence could prolong the OS and SAR of the patients as compared to other treatments. Furthermore, ELR after R0 resection indicated a worse prognosis than those with LLR, and TLR can be considered an independent prognostic factor for OS and SAR. If substantiated in a larger, multicenter study, the observations from this pilot study might provide the rationale to develop individualized surveillance programs for the patients at higher risk, providing an earlier diagnosis and better second treatment chance in the case of a recurrence.

## Data Availability Statement

The raw data supporting the conclusions of this article will be made available by the authors, without undue reservation.

## Ethics Statement

The studies involving human participants were reviewed and approved by the institutional review board of Sun Yat-sen University Cancer Center. Written informed consent to participate in this study was provided by the participants’ legal guardian/next of kin. Written informed consent was obtained from the individual(s) and minor(s)’ legal guardian/next of kin, for the publication of any potentially identifiable images or data included in this article.

## Author Contributions

YL, TG, and DH collected the patients’ data. YL and TG did the data analysis. All authors designed the study, and YL, TG, and DH finished the original manuscript. All authors contributed to the article and approved the submitted version.

## Funding

This study was supported by grants from the National Natural Science Foundation of China (no. 81902736).

## Conflict of Interest

The authors declare that the research was conducted in the absence of any commercial or financial relationships that could be construed as a potential conflict of interest.
