# Supplementary material for: Time to Local Recurrence as a Predictor of Survival in Patients With Soft Tissue Sarcoma of the Extremity and Abdominothoracic Wall
Source: Front Oncol. 2020 Nov 4;10:599097. doi: 10.3389/fonc.2020.599097 (PMC7672181; doi:10.3389/fonc.2020.599097)
Supplement: Supplementary file 1 [file Presentation_1.pptx]

## Slide 1
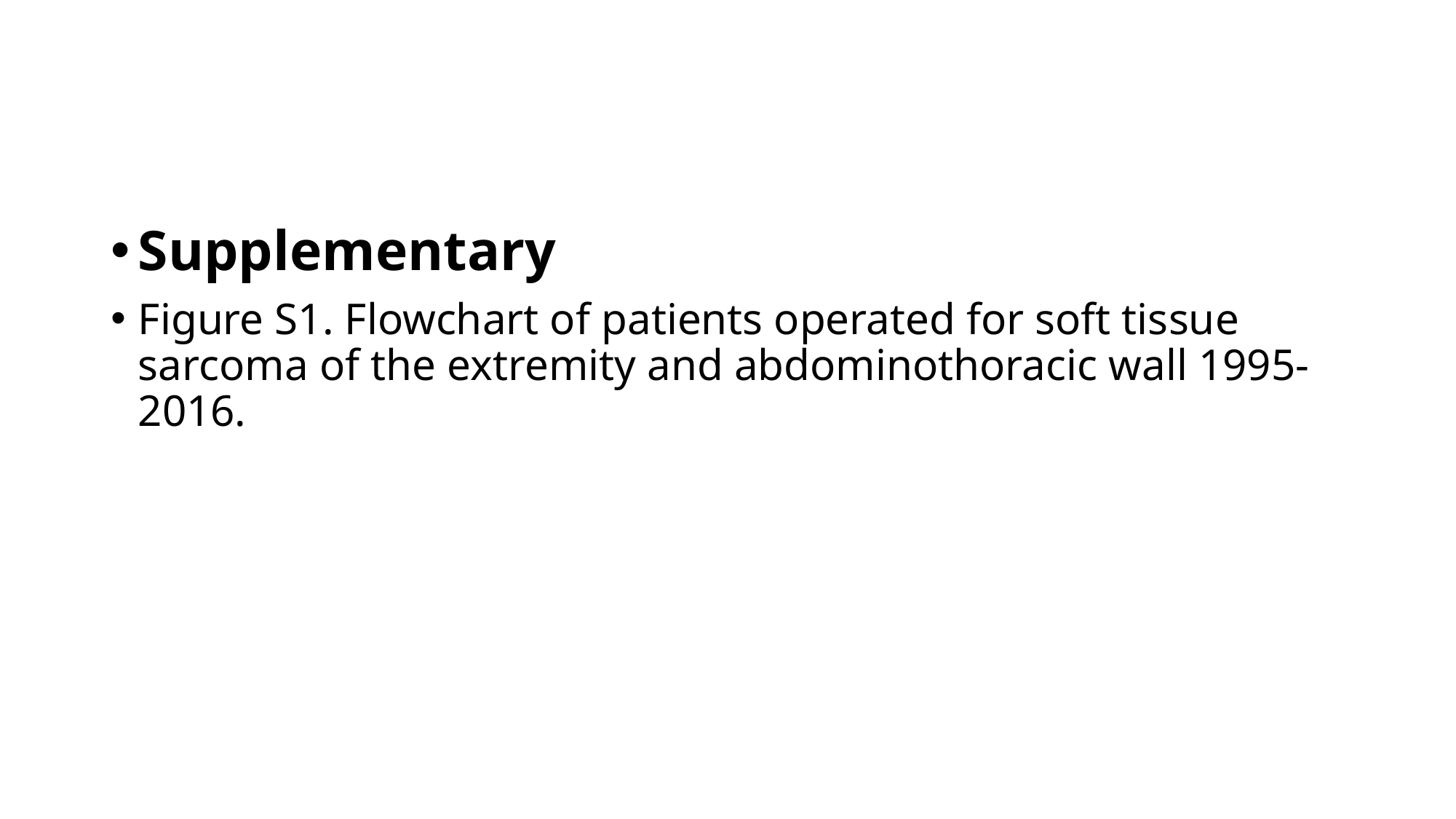

Supplementary
Figure S1. ﻿Flowchart of patients operated for soft tissue sarcoma of the extremity and abdominothoracic wall 1995-2016.

## Slide 2
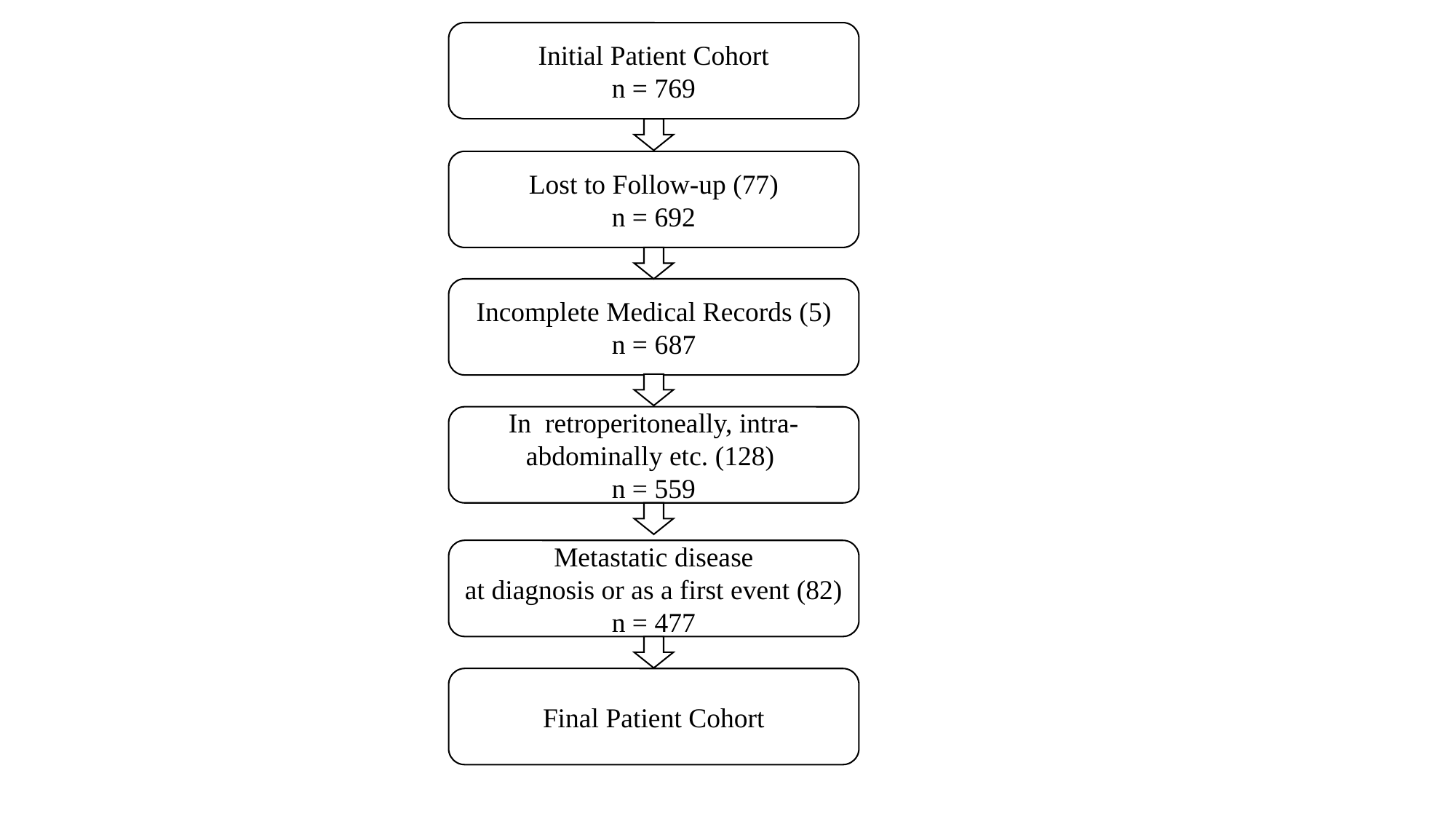

Initial Patient Cohort
n = 769
Lost to Follow-up (77)
n = 692
Incomplete Medical Records (5)
n = 687
In retroperitoneally, intra-abdominally etc. (128)
n = 559
Metastatic disease
at diagnosis or as a first event (82)
n = 477
Final Patient Cohort
